# Evaluating the Quality and Impact of Online Patient Forums in Genomic Data Governance

**DOI:** 10.1111/hex.70789

**Published:** 2026-07-30

**Authors:** Apondo Eric, Schickhardt Christoph, Andrea Züger, Eva C. Winkler, Mehlis Katja

**Affiliations:** ^1^ Faculty of Medicine, Institute for Medical and Data Ethics Heidelberg University Heidelberg Germany; ^2^ National Center for Tumor Diseases (NCT) Heidelberg Germany; ^3^ German Cancer Research Center (DKFZ) Heidelberg Germany; ^4^ Institute of the History, Theory and Ethics of Medicine Justus‐Liebig‐University Gießen Germany

**Keywords:** evaluation, genomic data archive, governance, health policy, online deliberative forums, patient involvement

## Abstract

**Background:**

There is consensus that patients' perspectives should be considered in decisions about health data. Deliberative forums (DFs) have become a common tool for patient involvement (PI) in health policy development. However, translating deliberative outcomes into policy decisions poses challenges. There has recently been interest in conducting DFs online, yet few online DFs have been evaluated for quality or policy impact. We evaluated a series of online DFs that were conducted to explore how patients can be involved in the governance of a genomic data archive (GDA), the German Human Genome‐Phenome Archive (GHGA).

**Methods:**

We conducted two online DFs and a follow‐up dialogue event with members of the cancer and rare diseases (RD) communities in Germany (*n* = 26). Evaluation was conducted using three approaches: (1) A pre‐/post‐survey that assessed the knowledge and opinions of the participants before and after the forums; (2) the OECD questionnaire for deliberative processes, which evaluates the design of the DFs and the deliberative experience from the participants' perspective, and (3) assessment of the pathway to impact of the DFs in terms of which deliberative outcomes the management of the GHGA committed to acting on as documented in a white paper, and which of these, at the time of writing, have been acted upon, or implemented as its governance policy.

**Results:**

Eighteen participants (69%) completed the survey on knowledge and opinions. A Wilcoxon Signed‐Rank Test indicated a statistically significant knowledge gain for all items in the knowledge category (*p* < 0.05). There was a significant change in one of the opinion items in the survey. A total of 24 participants (96%) completed the OECD survey; 21 participants (87.5%) reported that the deliberations and their outcome met their expectations. The participants made 14 recommendations for the PI policy of the GHGA. The GHGA management committed to 13 of the 14 recommendations from the DFs. These recommendations were incorporated into a white paper proposing a policy framework on PI in the governance of the GHGA. We identified 7 recommendations from the white paper that have been acted upon by the GHGA or implemented as part of its policy.

**Conclusions:**

Online DFs can achieve high deliberative quality and meaningfully influence decision‐making about governance and policy development. Our findings demonstrate the feasibility of online patient participation in health data governance and offer empirically grounded insights for the translation of patient and public deliberation outcomes into policy‐making and governance processes.

## Patient or Public Contribution

Two patient co‐researchers were involved in all phases of the study as part of the study team. Before their recruitment, they were informed about the study's goals, its planned duration, their roles, and the expected time demands for each role. They were involved in the following tasks: development of the study protocol, development of a recruitment strategy, development of the discussion guide and information materials for the participants, development of the programme of the DFs, including decisions about the length of the individual sessions, as well as the number and the length of the breaks. They were also involved in preparing the manuscript of the white paper. The patient co‐researchers were remunerated for their work in the study.

## 1 Introduction and Background

Patient involvement (PI) has been identified as an important ethical and societal requirement of genomic research and the development of policy involving genomic data [1, 2]. There is a broad consensus that patients' perspectives should be actively sought and considered in decisions about the collection, use, and governance of health data [3]. Deliberative forums (DFs) have become an increasingly common tool for involving stakeholders, including patients, in health policy development [4, 5], where a wide range of perspectives about new, unfamiliar, or ethically complex issues are expected to be discussed or raised by participants [6]. In DFs, participants are informed extensively about relevant topics before discussions. This occurs in written form, through presentations by experts, or both [7]. DFs emphasize dialogue between participants to enable collective weighing of points of view, including the trade‐offs associated with particular solutions [8]. They have been shown to improve the relevance of decision‐making processes [7, 8]. With the development and wide‐scale use of videoconferencing platforms since the COVID pandemic, there has been increased interest in online qualitative research methods, including DFs [9, 10].

There are guidelines for conducting face‐to‐face DFs [11, 12, 13]. However, there has been debate as to whether the standards of face‐to‐face DFs, such as providing adequate information and a high quality of deliberation based on an informed weighing of various perspectives, can be achieved online [14, 15]. Moreover, although DFs can be of high quality [6] and be successful even if they don't achieve the intended impact in terms of policy [16, 17], there is broad consensus that DFs conducted to develop policy should include relevant stakeholders and, *ideally*, have a demonstrable impact on decision‐making and policies [18]. In the literature on political participation, there are other examples of online democratic forums [19, 20]. However, overall, there are very few examples of online DFs, and even fewer that have been evaluated [21, 22]. Studies show that many DFs, including those conducted face‐to‐face, do not have demonstrable impact [23, 24], and that in most cases, their outcomes are not taken up in what becomes ‘stated policy’ [25, 26]. There is a ‘gap between deliberative outcomes […] and measurable policy impact’ [27]. This is partly due to practical challenges that impede the translation of patient perspectives into policy [28]. In addition, little is known about how participants experience online DFs [29]. To bridge these knowledge gaps, research that focuses on the translation of DF outcomes into policy is needed [30].

This article reports on how we conducted two online DFs with members of the cancer and rare diseases (RD) communities in Germany in the PaGODA study (**Pa**tient Involvement in the **G**overnance of an **O**mics **D**ata **A**rchive), consulted with the management of a genomic data archive (GDA), the German Human Genome‐Phenome Archive (GHGA), to translate the outcomes of the DFs into a white paper with recommendations for the PI policy of the GHGA, and evaluated the quality of the DFs and the translation process. The PaGODA study explored German participants' perspectives concerning the role of patients in the governance of GDAs in general, and how these recommendations could be taken up as part of the governance framework of the GHGA specifically. The white paper [31], as well as the results of the deliberations [32], have been previously published.

This article contributes to the literature by (a) presenting the results of the empirical evaluations of online DFs in the context of governance of genomic data, (b) offering suggestions on best practices for conducting online DFs based on our evaluation, (c) tracing the pathway to impact of the DFs by examining which deliberative outcomes the management of the GHGA committed to implementing in a white paper, and which of these commitments had, at the time of writing, been incorporated into the GHGA's governance policy, and (d) offering lessons for integrating PI into governance of data‐sharing infrastructures.

## 2 Methods

### 2.1 Definitions

In this paper, ‘patient involvement’ (PI) refers to ‘meaningful and active’ collaboration with patients and their representatives [33]. ‘Governance’ refers to ‘interactions among structures, processes, and traditions that determine how power and responsibilities within an organization are exercised, how decisions are made, and how […] stakeholders have their say’ [34] (These definitions of ‘patient involvement’ and ‘governance’ were used in a previous publication on the results of the PaGODA study [35]). The use of the term ‘policy’ in the health setting is multifaceted, and the definitions by different health organizations and institutions vary [35, 36, 37]. The Centers for Disease Control and Prevention (CDC) defines it as the ‘regulations, procedures, administrative actions, incentives, or voluntary practices of […] institutions and organizations, at national, state, or local level’ [36]. In this paper, we present a deliberative process to develop a PI policy for the governance of a GDA; we use the term ‘policy’ to describe the officially documented or stated procedures that establish if and how patients or patient representatives can have impactful roles in the governance of GDAs (or similar infrastructures). In this sense, policy officially and explicitly delineates elements of governance.

### 2.2 PaGODA‐Study Design and Steps to the Implementation of the Findings

In this section, we describe the PaGODA study before describing its evaluation in the next section. A description of the rationale for the methodology of the PaGODA study, the study design, as well as comprehensive descriptions of the educational materials, the discussion guide, sampling, and recruitment procedures have been previously published [32]. After an extensive literature review to identify possible research methods, we chose the qualitative method, and specifically DFs, to explore the diverse perspectives and complex ethical aspects that we expected to be raised by the participants, and to allow for collective weighing of views and potential trade‐offs [38]. Two patient co‐researchers were involved in all phases of the study (see statement on patient contribution above). The study protocol was approved by the Ethics Commission of the Medical Faculty of the University of Heidelberg (Ref. S‐061/2022). All participants signed informed consent before participation.

Purposive sampling was used to recruit participants nationwide in Germany. We sent invitations via the email mailing lists of the 3 main organizations for patients with RDs and cancer in Germany: *Allianz Chronischer Seltener Erkrankungen, Haus der Krebs‐Selbsthilfe and Bundesarbeitsgemeinschaft Selbsthilfe*. A total of 26 participants were recruited for the study. Two 7‐h online semi‐structured DFs were conducted in 2022 on July 9th (10 participants) and 22nd (16 participants). For each DF, the participants were divided into two small groups, which were stratified according to sex, age, general disease entity, and level of education [32]. Each participant was compensated with 100 Euros for participation in the study.

**Figure 1:**
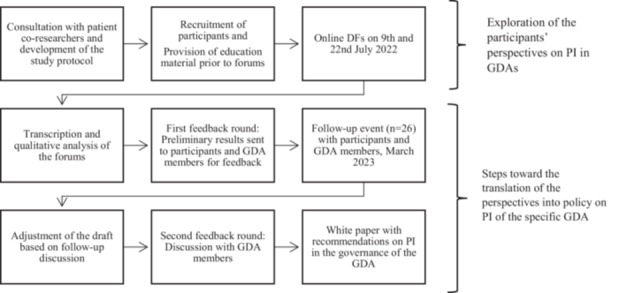
Overview of the PaGODA study, previously published in a white paper detailing the PI policy of the GDA [37].

The study had a sequential design [39] that included feedback rounds to iteratively build consensus not just between the DF participants, but also between the participants, GHGA management and the other stakeholders in the GHGA (board of directors, researchers and the team leaders). Figure 1 shows an overview of the study design and steps. Each DF was divided into two main sessions: a morning session consisting of small group discussions, and a plenary session in the afternoon. There were two 10‐min breaks in the morning session and a lunch break from 1 to 2 PM. The small groups in the first and second forums consisted of five and eight members each, respectively. The agenda of the DFs is shown in Table 1. In the plenary sessions, the participants summarized the discussions from the small groups and formulated recommendations that would be presented to the management of the GHGA. These recommendations in turn informed the discussion in a 2‐h follow‐up dialogue event on 3rd March, 2023 between the participants and GHGA members; 17 of the initial DF participants and 9 GHGA team members (including management) attended this event (*n* = 26). For data security reasons, the online DFs and the dialogue event were conducted and recorded using the Webex videoconferencing tool, which was provided by the University of Heidelberg, where the study was conducted.

**Table 1:** Agenda of the deliberative forums.

Format	Time	Topic
Plenary session	9:00–9:10	– Welcome address – Introduction of the study team and moderators – Forum programme – General rules
	9:10–9:40	Expert presentation 1: Introduction to the GDA: Its development, roles, objectives, and goals
Breakout session	9:40–10:10	Introduction round in small groups Discussion, Theme Block 1: Consent and release of data for medical research
	10:10–10:20	Break
	10:20–11:20	Discussion, Theme Block 2a: Patient involvement in the governance of research data archives: General aspects
	11:20–11:30	Break
Plenary session	11:30–12:00	Expert presentation 2: Informed consent
Breakout session	12:00–13:00	Discussion, Theme Block 2b: Patient involvement in the governance of research data archives: Data use and access
	13:00–14:00	Lunch break
Plenary session	14:00–15:45	Presentation of the summaries of small group discussions and further discussion, especially of the themes not raised in the small groups. Formulation of recommendations to be presented to the GDA management.
	15:45–16:00	Feedback from participants End of deliberative forum

### 2.3 Evaluation of the Quality of the Deliberative Forums

Based on a review of the literature, we evaluated the quality of the online DFs using three approaches:

1. Knowledge levels and changes of opinions concerning the topics of deliberation [40, 41]: We designed a 10‐item knowledge and opinion questionnaire (Table 2) in which the participants self‐reported on their knowledge and opinions about PI in GDAs. The questionnaire was completed by the participants before and after the DFs.
2. The OECD questionnaire for evaluation of deliberative processes [13] (Table 5): In this questionnaire, the design of DFs and the deliberative experience of the participants are evaluated using such criteria as inclusiveness, representativeness, quality of facilitation, and equality of opportunities to speak. The questionnaire includes Likert items on a 10‐point scale as well as open‐ended questions.
3. Assessment of the pathway to impact of the DFs: This was partly evaluated in the OECD questionnaire (see 2, above, and Table 5, below), but also analyzed separately, by examining how many of the participants' recommendations the management of the GHGA committed to acting on as documented in a white paper, and which of these, at the time of writing, have been acted upon, or implemented as the governance policy of the GHGA.

**Table 2:** Knowledge and opinion questionnaire (the questions were answered on a 5‐point Likert scale).

Item	Statement	Category
1	I am well informed about patient involvement in governance	Knowledge
2	I understand the roles GDAs play in medical research	Knowledge
3	I understand the process through which patient samples become genomic data stored in a GDA	Knowledge
4	I understand how researchers get access to data in GDAs	Knowledge
5	If I were to agree to having my data stored in a GDA, I would trust that researchers will use my data according to the stipulations of my consent	Opinion
6	Governance of GDAs without patient involvement is sufficient	Opinion
7	Patients should be involved in the governance of GDAs	Opinion
8	I understand the roles that patients can take in the governance of GDAs	Knowledge
9	Patient involvement in the governance of GDAs is best achieved through patient representatives	Opinion
10	I am interested in actively participating in the governance of a GDA	Opinion

All questionnaires were completed electronically. The pre‐ and post‐questionnaires were sent to the participants via email. The OECD Questionnaire was completed on the LimeSurvey platform (www.limesurvey.org), for which each participant received a separate link after the DFs.

### 2.4 Data Analysis and Statistical Methods

Quantitative data from the pre‐ and post‐questionnaires were analyzed using descriptive statistics and the Wilcoxon signed‐rank test for paired comparisons to assess changes in self‐reported knowledge and opinions before and after the DFs. We used the Wilcoxon Signed‐Rank Test Calculator available on the Social Science Statistics website (www.socscistatistics.com/tests/signedranks/calculator/). We also calculated the *r*‐values to determine the effect size [42] (see Table 4). Missing data were excluded listwise when participants did not complete both pre‐ and post‐questionnaires. Items were treated as ordinal variables, and median values were calculated for all Likert‐scale items. For the OECD questionnaire, descriptive statistics (frequencies and medians) were used to summarize responses (see Table 5). Qualitative data from open‐ended survey responses were analyzed thematically.

## 3 Results

### 3.1 Participants

Twenty‐nine individuals accepted the invitation to participate in the DFs, and 26 took part in the forums (Table 3); 18 (69%) of the participants were female. Most of the participants were active members of a patient organization, and the largest age cohort (*n* = 11, 42%) was in the 50–59 years age bracket; 14 participants (53%) had a college diploma.

**Table 3:** Participants (previously published in [32]).

Total number who accepted the invitation	29
Dropped out prior to the forums	1
Did not participate due to illness	2
Forum participants	26
Male	8
Female	18
Disease category (more than one possible)	
Rare disease (RD)	10
Genetic predisposition for an RD	2
Cancer	7
Genetic predisposition for cancer	7
Relative of individual with RD	1
Representative of an RD patient group	1
Age	
18–29	1
30–39	1
40–49	5
50–59	11
60–69	6
70–79	2
Highest level of education	
Secondary school	6
Highschool diploma	6
College diploma	11
Doctorate	3
Member of a patient organization	
Yes	22
No	4

#### 3.1.1 Pre‐ and Post‐Questionnaire on Knowledge and Opinion

Eighteen of the 26 participants completed both the pre‐ and the post‐questionnaires assessing knowledge and opinion (response rate of 69%). Two participants completed only the pre‐questionnaire, two completed only the post‐questionnaire, four completed neither questionnaire.

**Figure 2:**
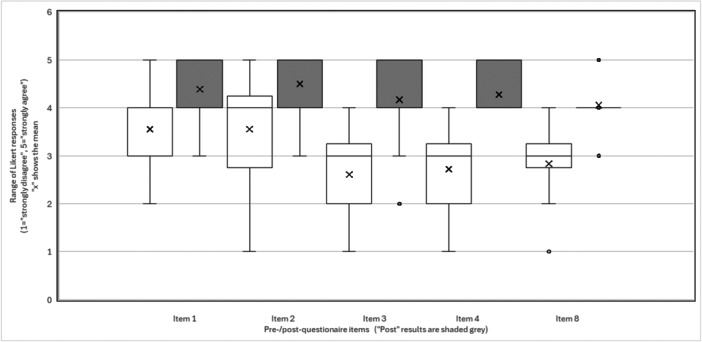
Knowledge pre‐/post‐questionnaire.

**Figure 3:**
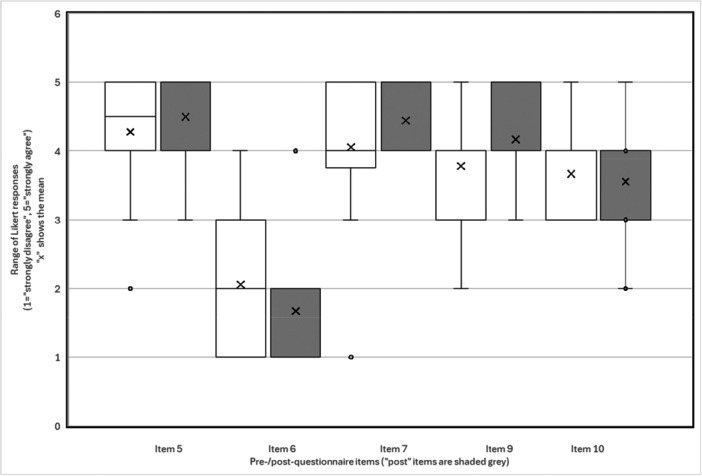
Opinion pre‐/post‐questionnaire.

A Wilcoxon Signed‐Rank Test was conducted to determine whether there was a statistically significant difference between the self‐reported knowledge and opinion scores before and after the DFs. Table 4 shows the median scores for the individual items before and after the DFs, as well as *Z*‐, *p*‐, and *r*‐values. The Wilcoxon Signed‐Rank Test indicated that the self‐reported knowledge levels for all five knowledge items were significantly higher after the DFs than before (*p* < 0.05) (Table 4 and Figure 2). The analysis of changes of opinion (see Figure 3 below) suggests that only changes in responses to Item 9 were statistically significant (*p* < 0.05) (Figure 3). The items with statistically significant changes all had a large effect size (*r*‐value > 0.5), suggesting that the DFs had a large effect on these results [42]. The detailed statistical analysis can be found in the Supporting Information.

**Table 4:** Statistical summary of the analysis of the knowledge and opinion questionnaires.

Category	Item	Median		*Z*	*p*‐value	*r*‐value (effect size)
		Pre‐	Post‐			
Knowledge	1	4	4	−2.99	0.0005	0.9
	2	4	5	−2.64	0.002	0.8
	3	3	4	−3.43	0.00003	0.9
	4	3	4	−3.43	0.00003	0.9
	8	3	4	−3.37	0.00006	0.9
Opinion	5	4,5	5	−0.79	0.258	—
	6	2	2	2.20	1.000	—
	7	4	4	−1.56	0.094	—
	9	4	4	−2.04	0.031	0.7
	10	4	4	0.44	0.734	—

*Note:* Significance level *p* = 0.05; the results shaded grey were not statistically significant.

#### 3.1.2 OECD Questionnaire

Twenty‐four of the participants completed the OECD participant questionnaire (response rate of 96%). The OECD evaluation framework evaluates three broad aspects of deliberative processes: (a) process design integrity, (b) the deliberative experience, and (c) pathways to impact. As recommended by the authors of the OECD questionnaire [13], we left out aspects of the original questionnaire that were not relevant to the specific DFs we conducted, or to the participants. Table 5 below shows a summary of the participants' questionnaire responses. For clarity and space reasons, the individual items have been summarized, and for the Likert items, only the medians are shown. The detailed summary of the OECD questionnaire can be found in the Supporting Information.

**Table 5:** Results of the OECD participant questionnaire.

**A. Process design integrity**	
**Criteria 1: Suitable design**	
Do you think the length of the process was appropriate?	Yes: 14 (70%) No, too short: 5 (25%) No, too long: 1 (5%) Not sure: 1 (5%)
If you consider the process needed more time, how much more?	1 day more: 2 At least one day more: 3 At least 2 days: 1
If you consider the process needed more time, how would you use the extra time?	Presentations from the experts invited: 1 Presentations from other experts: 2 General discussions: 10 Discussing the recommendations: 7 More breaks: 2
Do you think the time was well used to arrive at the final recommendations? (0 means ‘not at all’, 10 means ‘extremely well used’)	Median: 7
How did you experience the balance between time spent in small group discussions and in plenary?	Too much time in small groups, too little in plenary: 2 (11%) Too much time in plenary, too little in small groups: 5 (26%) The balance was just right: 12 (63%)
**Criteria 2: Representativeness and inclusiveness**	
How many of the other members had different views compared to your own?	None: 2 (9%) A few: 9 (42%) About half: 4 (19%) Most: 4 (19%) I don't know: 2 (9%)
Did you feel there were any groups **not** represented?	Yes: 4 (19%) No: 12 (57%) No answer: 5 (23%)
**B. The deliberative experience**	
**Criteria 1: Neutrality and inclusivity of facilitation**	
To what extent do you feel the facilitators were neutral or biased? (0 means ‘completely neutral’; 10 means ‘very biased’)	Median: 1
**Criteria 2: Breadth, diversity, clarity and relevance of the evidence and stakeholders**	
Do you feel the information resources provided to you to help (0 means ‘not sufficient’, 5 ‘just right’ 10 ‘too much information’) discussions were narrow or broad?	Median: 5
Do you feel that the information resources provided were neutral, with fair and diverse viewpoints represented?	Median: 5
Did you find the evidence that was presented by the speakers easy or hard to understand?	I understood the presentation well right from the beginning: 17 (89%) The presentations were difficult to understand at the beginning, but got easier as the forums progressed: 2 (10%) The entirety of the presentations was difficult to understand: 0 I don't know: 0 No answer: 0
**Criteria 3: Quality of judgement**	
Do you feel that the issue was discussed from a variety of perspectives (e.g., considering underlying issues, existing structures, trade‐offs, values, etc.)? (0 means ‘limited number of perspectives’, 10 means ‘wide range of perspectives’)	Median: 7
Do you feel that most members were providing justifications and explanations for their opinions? (0 means ‘most never provided justifications’, 10 means ‘most provided justifications’)	Median: 8
**Criteria 4: Perceived knowledge gains by members (answers on a scale of 0 to 10, 0 means ‘not at all’, 10 means ‘to a great extent’).** **To what extent do you feel that:**	
Your understanding of the issue became clearer throughout the process?	Median: 9
You gained more arguments and perspectives to support your own opinion about the issue?	Median: 7
You understood the arguments, perspectives and concerns of others?	Median: 9
Your understanding of others’ opinions of the issue became clearer through this process?	Median: 7
**Criteria 5: To what extent do you feel that you were well informed about the following subjects? (answers on a scale of 0 to 10, 0 means ‘not at all’, 10 means ‘to a great extent’)**	
Informed consent as pertains to genomic (research) data	Median: 10
Data sharing	Median: 10
Governance structures of genomics data archives	Median: 8
**Criteria 6: Accessibility and equality of opportunity to speak. To what extent do you feel (0 means ‘absolutely disagree’, 10 means ‘absolutely agree’):**	
You had a fair number of opportunities to speak?	Median: 10
Other members had a fair number of opportunities to speak?	Median: 10
All members were heard equally?	Median: 10
Some members dominated the small group discussions?	Median: 7
You and your views were heard?	Median: 10
**Criteria 7: Respect and mutual comprehension**	
To what extent do you feel that fellow members respected what you had to say, even when they didn't agree with you?	Median: 9
**C. Pathways to impact**	
Imagine you are the decision‐maker that convened this process. Would you implement the recommendations the deliberative process produced?	Yes, all of them: 0 Yes, most (> 75%): 14 Yes, about 50%: 2 Yes, some (25%–50%): 2 No: 0
Does the outcome of the deliberative process match your expectations?	Yes: 21/24 (87.5%) No answer: 3 (12.5%)
What form should patient involvement in genomic data archives take?	‐Involvement of patient representatives in existing boards: 14 ‐Establishing separate patient advisory boards: 8 Other: 1

*Note:* All percentages shown are rounded off and may therefore not total to 100% for each item.

##### 3.1.2.1 Participants' Survey Responses on the Design of the Forums

Twenty‐one participants (87.5%) reported that the outcome of the deliberations matched their expectations. Most of the participants (70%) felt that the duration of the DFs was appropriate. They were satisfied with how time was allocated to reach the final recommendations, with a median score of 7 out of 10. In addition, 63% of the respondents found the balance between the time spent in small groups and in plenary appropriate. Of those who were not satisfied, most (five participants) would have preferred more time in small group discussions. Most participants felt that the composition of the DFs was diverse and that relevant groups were represented; only four participants (16%) felt that some relevant groups were not represented. Most participants (*n* = 17, 81% of respondents for the item) reported that many of their fellow participants had opinions that differed from their own, which supports the participants' reports on their perceived diversity of the composition of the DFs.

##### 3.1.2.2 Participants' Survey Responses on the Deliberative Experience

Concerning the breadth, diversity, and relevance of the information provided, most participants reported that the information provided before and during the DFs was adequate, neutral, appropriate and presented from an appropriate range of perspectives (median score of 5, corresponding to the sentiment ‘just right’ on a scale of 0 to 10 [‘not sufficient information’ to ‘too much information’]). A total of 17 of the 19 respondents on the questionnaire item (89%) reported that they understood the presentations right from the beginning. The participants reported that they were adequately informed on the topics of informed consent (median: 10, with answers on a scale of 0 to 10, 0 meaning ‘not at all’, 10 meaning ‘to a great extent’), data sharing (median: 10), and governance structures of GDAs (median: 8). Moreover, most participants reported that the topics were discussed from a variety of perspectives (median: 7) and that other participants gave sufficient justifications for their opinions (median: 8).

The participants reported having gained knowledge from the deliberative process, responding that the issues discussed and the perspectives of others became clearer to them during the process (median: 9). Moreover, they reported having gained additional arguments to support their own perspectives (median: 7). Most participants reported that they and other participants had a fair number of opportunities to speak (median: 10), that all members were heard equally (median: 10), and that mutual respect was shown even when there was disagreement (median: 9).

##### 3.1.2.3 Pathways to Impact

Twenty‐one participants (87.5%) reported that the outcome of the deliberative process matched their expectations. An open‐ended question in the OECD questionnaire about whether the DFs met the participants' initial expectations was answered as follows by one participant:

Yes [the DFs met my expectations]. The agenda clearly matched the issues that were raised in the invitation and […] gave answers to these issues. In addition, information and knowledge regarding the structure of genomic databases and patient involvement were provided and/or expanded upon.

Another participant commented on the information given, and the nature and framing of the issues that were discussed:

Going in, I had expected there to be more fully formed ideas that would then be discussed. However, the questions posed during the workshop were very open‐ended and allowed for a great deal of freedom in how they were answered; all topics were discussed in an open manner.

Seventy‐two percent of the respondents reported that if they had organized the online DFs, they would implement the recommendations reached during the process. Of these, 75% responded that they would implement most of the recommendations. In this regard, there was an open‐ended question in the questionnaire asking the participants what their understanding was of the steps that would be taken after the DFs:

In your understanding, what will the organizers of the event do with your recommendations?

The responses of the participants corresponded with what was discussed during the forums and showed a good understanding of the goals of the study:

They will hopefully use the recommendations to develop a governance structure for the GHGA to achieve or contribute to a broad public acceptance of genomic data archives.

I expect that the recommendations will be analyzed and the themes that were discussed will be further discussed with the current management of the GHGA.

The recommendations from the DFs (Table 6) were the basis of a follow‐up event on 3rd March 2023, in which 17 participants and 9 GHGA team members took part. The GHGA members included a member of the board of directors, the chief administrative coordinator, a data manager, the chief communications officer, and members of the ethics team. The aim of this event was to bring the DF participants and GHGA members together to discuss the DF recommendations. Moreover, the participants were able to further discuss which form PI should take in the GHGA, a topic on which there had been disagreement during the DFs: During the DFs, some participants had favoured the establishment of a separate patient advisory board (PAB), while others felt that it would be sufficient to have the patients integrated into already existing working groups and boards within the GHGA (Table 5, Item C). After discussion in the follow‐up event, the participants supported the establishment of a separate PAB.

**Table 6:** Consensus recommendations from the deliberative forums.

**Recommendations of the participants for the implementation of PI in GDAs**
1. **Areas of GDA governance in which participants can be involved**
a. Outreach activities and communication, including actively establishing avenues for patients to meet and exchange ideas with researchers and other stakeholders
b. Data access: Developing ways to make data access practices of GDAs more transparent to patients or their representatives^a^
c. Advising data subjects and other stakeholders, including researchers
d. Create a pool of patients who can be contacted for consultations in the future
e. Developing information material for patients that is patient‐friendly
f. Keeping patients updated about research through apps and newsletters
g. Informing patients about how they can be involved in research and jointly exploring possibilities for PI in research with pharmaceutical companies
2. **Facilitation: Resources necessary for meaningful PI in the governance of GDAs**
a. Continuous training and information about GDA projects
b. Financial compensation and acknowledgement of patients’ work
c. Logistical support, e.g., parking, access to scientific journals, information materials
3. **Representation: What form PI should take in GDAs and its legitimacy**
a. Recruitment of patients as representatives to form a patients' advisory board (PAB)
b. No prior formal training necessary to represent other patients
c. Good listening and communication skills of patient experts
d. Potential representatives should be committed to representing not just themselves; they should have a group perspective

^a^ This recommendation could not be taken up in the white paper with proposals for the governance policy of the GDA due to limitations in the functionality of the archive at the time. All other recommendations were taken up in the white paper.

The participants made 14 final recommendations for the implementation of PI in the governance of the GHGA (Table 6). Of these, one recommendation (Recommendation 3a, discussed above) was made during the follow‐up event. The recommendations were presented to the management of the GHGA, and 13 of the recommendations were taken up in a white paper proposing a policy concept for PI in its governance. Therefore, the actual steps that were taken after the DFs reflected the participants' expectations (see Figure 1).

## 4 Discussion

This paper presents results of evaluations of the quality and impact of online DFs that were conducted as part of a participative process towards the development of policy on PI in the governance of a specific GDA. Overall, we find that online DFs can serve as a feasible and impactful approach to PI. This study adds to a small body of literature on the evaluation of online approaches to PI. Our results offer important considerations for the emerging field of online public deliberation and for PI in the governance of genomic data. First, we present aspects that are relevant to establishing best practices of conducting online DFs. Second, we discuss how our findings may contribute to efforts to successfully translate patient perspectives on genomic data from DFs into policy, and thus to achieve demonstrable impact. Finally, we discuss which recommendations from the white paper have been acted upon by the GHGA, or implemented as part of its policy.

### 4.1 Best Practices for Conducting Online DFs

The participants' positive feedback on the clarity and relevance of the information provided before and during the forums, as well as their satisfaction with the diversity and length of the DFs, and the responses from the knowledge pre‐/post‐survey are important in the context of online DFs; they show that basic requirements of DFs, such as providing adequate information and fostering dialogue to enable collective weighing of opinions [43] can be achieved in online DFs. The participants also assessed the design of the DFs favourably: most were satisfied with the diversity of the DFs, their length and how time was allocated. Previous similar studies have mentioned ‘Zoom exhaustion and fatigue’ [44, 45] when engaging with stakeholders remotely. There have also been discussions in the literature concerning how long online qualitative DFs should last, and it has been suggested that 90‐min slots allow for sufficient discussion without being exhausting for the participants [46]. We learned that an agenda that interspersed presentations, Q&A sessions, small group discussions, plenary, and breaks made it easier for the participants to stay engaged throughout the online DFs, although they lasted several hours. The responses concerning the length of the small group sessions suggest that, in planning such forums, care should be taken to plan sufficient time for small group discussions.

Concerning the composition of the DFs, we recruited a heterogeneous group of patients from various patient groups and (self‐help) organizations nationwide in Germany [32]. Moreover, the recruited participants were further stratified according to demographic characteristics, which were considered when constituting the small groups. This contributed to a lively discussion, with participants reporting that they heard viewpoints that differed from their own. Consequently, we recommend this stratification strategy in constituting online DFs. Appropriate group size has been mentioned in the literature as a factor that ensures that all participants have a chance to speak [15]. Previous similar studies have recommended 4–8 participants as being ideal for small group discussions [46], and the number of participants in the small groups in this study (five each in the first online DF and eight in the second) enabled a lively exchange with all participants being engaged in the discussions. This size also made it possible for the participants and the moderators to see all participants on the screen at the same time, an aspect that has been identified in the literature as important for online discussions [45]. One of the challenges of online group communications that has been mentioned in the literature is that participants may be less likely to elaborate on their opinions [47, 48]. We did not observe this in this study; the participants reported that other participants explained their opinions adequately.

Participants evaluated the online forums positively and reported increased knowledge and a deeper understanding of the governance issues under discussion. Survey responses and the substantive recommendations developed during the forums and follow‐up event suggest that the informational materials and the presentations during the DFs provided a sufficient basis for informed and meaningful participation. The deliberations resulted in concrete and clearly articulated recommendations across multiple governance domains. The online DFs therefore met the standards that have been proposed for quality face‐to‐face deliberation, such as those identified by Elstub and Carson of (a) participants being well informed about the topic, (b) discussing varied perspectives, so that they (c) ‘arrive at a public judgement (not opinion) about “what can we strongly agree on?”’ [14].

Concerning the moderation of the DFs, on the one hand, the participants felt they and their fellow participants had a fair number of opportunities to speak, and that all members were heard equally. On the other hand, many also reported feeling that some members dominated the small group discussions (Table 5, Item B, Criteria 6). This suggests that in some instances, moderation was not effective. It has been previously observed that compared to face‐to‐face discussions, the moderator in online group discussions needs to have a ‘more active and directive role’, and has ‘an increased responsibility for the active inclusion of all meeting participants’ [45]. A factor that may have made the moderation challenging in some instances of the DFs in this study is the level of participatory experience that some participants had compared to others: some of the participants were not only active members in patient organizations, but also had leadership positions in these organizations and had previously been involved in discussions with other experts in the German health sector at the local, regional and even the national level. With this background, these participants likely had more to say and were more articulate on the issues than their counterparts. Moreover, the complexity of the topic may have led these participants to direct their comments at the moderators, rather than each other, possibly because they felt the moderators would understand them better. This tendency of online forum participants to communicate with the moderators instead of with their fellow participants has been previously reported [49]. Since diversity is important in DF discussions, it is not desirable to constitute small groups in online DFs such that all the participants have the same background or level of experience on the topics being discussed. However, moderators should consider and be trained in strategies for meeting this challenge during online forum discussions.

The OECD questionnaire proved to be a useful tool for evaluating online DFs. We suggest that the questionnaire be adjusted to include factors relevant to the online setting, such as feedback about online tools and connectivity, as well as feedback concerning the number of participants. Moreover, digital technology can be used to more accurately evaluate the quality of discussions in online DFs by measuring, for example, how frequently the participants participate, and how long the participants talk vis‐à‐vis the facilitators.

### 4.2 Impact: Towards Translation of Patient Perspectives Into Governance Policy

Our study is one of very few in the literature that describes and evaluates an online DF process in the case of genomic data and research. We found one example in the literature of an *online* DF conducted to explore public perspectives on access to health data [50]. The study has several methodological similarities to the PaGODA study in terms of strategies deployed to educate participants (written and expert presentations), compensation of participants, number of sessions, structure of the online DFs with division into small and large groups, and the strategy for analyzing the DFs [32]. However, the authors did not aim to translate the perspectives explored into policy, nor did they evaluate the DFs. We cannot, therefore, draw any comparisons to our study pertaining to evaluation.

There are examples in the literature of *face‐to‐face* DFs conducted to inform policy on genomic data with learnings that are relevant to online DFs: O'Doherty and colleagues conducted a face‐to‐face DF with 25 residents of British Columbia in Canada to inform guidelines for contacting individuals for participation in a biobank [28], while Molster and colleagues conducted a DF with 16 residents of Perth, Western Australia, on the ethical, legal and social issues related to biobanking and the use of genomic data [30]. Methodological challenges that were identified in the face‐to‐face DFs included the challenge for the recruitment process to include citizens who lived far away from where the studies were conducted [30].

The management and other stakeholders in the GHGA committed to all but one of the 14 participants' recommendations, which were then documented in the white paper. Four main factors may have contributed to the high uptake of this study's deliberative outcomes into the white paper with recommendations for the policy of the GHGA: (a) The functional capabilities of the GHGA and its limits, as well as the challenges involved in creating or changing policy, including the legal constraints, were clearly communicated to the participants. As a result, the participants had a realistic picture of the level of impact they could expect to achieve from their participation in the DFs and therefore made recommendations that fit within the existing legal and resource framework of the GHGA. The importance of making the goals and limits clear to the participants at the outset of the deliberative process has been highlighted in the literature [51]. O'Doherty and colleagues also identified legal challenges and other already existing structures, such as research ethics boards, as possible obstacles to translation of recommendations, or to the change of existing policies [28]. (b) Together with the co‐researchers, we carefully considered aspects that would be relevant for the long‐term implementation and sustainability of PI in the governance structures of the GHGA at the design stage. Questions about these aspects were extensively discussed with the patient co‐researchers and were incorporated into the discussion guide that was used during the forums. The factors identified by the participants included continuous training of patient representatives, financial remuneration, and logistical support (see Table 6). Discussing the factors necessary for implementation was especially important in ensuring timely, constructive, and productive discussions during the DFs, and in building a bridge between the expectations of the participants on the one hand, and what the GHGA management had to do to fulfil those expectations, on the other. (c) The patient co‐researchers, who were involved in all phases of the study, were key in ensuring the format and content of information material were suitable for the participants. Through their suggestions and critiques, they helped keep the study's aims, design, and execution focused on the participants' needs and expectations. (d) We had a multidisciplinary research team, which included social scientists, philosophers, ethicists, and medical doctors. Moreover, GHGA decision‐makers and stakeholders, including the management level, cooperated with the study team closely during all stages of the study, especially during the consultation phases of the study, as well as in the follow‐up event. Previous studies have shown that many deliberative processes aimed at informing policy development did not result in useful recommendations due to a lack of a clear link between the participants and decision‐makers who are responsible for implementation [26].

### 4.3 Impact of the White Paper on the Governance of the GHGA

At the time of writing, we identified 7 recommendations that have been acted upon by the GHGA or implemented as part of its policy. These are the recommendations 1a, 1c, 1 d, 1e, 2a, 2b and 2c in Table 6. Since the white paper was published, there have been efforts by the GHGA to establish avenues for patients and GHGA members to meet and exchange ideas. For example, the recommendations from the DFs have led to outreach activities and focus groups where patients' views about aspects of use of genomic data have been sought, such as designing a patient website and developing strategies for communicating with patients about how their data are used and shared for research [52]. As recommended in the white paper, the participants of these focus groups were financially compensated for their participation, and the GHGA covered travel and lodging expenses. A member of the outreach and communications team has informed us that since the focus groups were conducted, the participants have been regularly informed about the progress on the development of the patient website and other GHGA activities via email or via the GHGA online newsletter, as was recommended in the white paper. The patient website will be active in June 2026. Through the DF and subsequent events, and with the consent of the participants, the GHGA is growing the number of patients it can contact in case it needs patient input on issues of governance in the future.

Unfortunately, the establishment of the PAB, which was one of the key recommendations in the white paper that was committed to by the management of the GHGA, has not been implemented in the governance structure of the GDA. The recommendations continue to be considered in ongoing discussions about PI policy implementation in the GHGA.

## 5 Limitations

This evaluation of the PaGODA study has a few notable limitations, the first being that the study team both conducted and evaluated the online DFs. Ideally, such evaluations should be conducted by an independent team to reduce bias [13]. Further, this study relied on self‐reporting for assessing knowledge gains and opinion shift. We had initially considered testing the knowledge of the participants with questions on various topics about GDAs and data sharing. However, after consultation with the patient co‐researchers, we decided against it, as it would have been too taxing for the participants, considering the breadth of the study and the significant investment in time and energy that it already demanded of the participants. In addition, although the pre‐ and post‐survey data showed changes in knowledge and opinion, it did not explain exactly which aspects of the DFs contributed to these changes (written information, presentations, small group discussions, or plenary discussions).

Not all participants completed the questionnaires. Technical difficulties may have contributed to this. Unfortunately, the participants were not provided with a quick method of reaching the organizers in case they had questions concerning the questionnaires, other than via email. Further, having only digital feedback from the participants via the questionnaires may have excluded more spontaneous feedback from the participants that may have been received from a discussion. Although conducting the forums online made participation easier for individuals who may otherwise not have been able to travel, it also excluded individuals who did not have access to video conferencing technology. Moreover, we recruited the participants through patient organizations in Germany, meaning that patients who were not members of the patient organizations were not reached and could therefore not take part in the DFs.

O'Doherty and colleagues [28] advise care in attempting to generalize the findings from DFs, depending on the participant population. In the case of the PaGODA study, the participants were members of the cancer and RD communities in Germany. As such, recommendations from the online DFs may present a narrow view specific to patients from these communities, and exclude other patients and the general public, limiting the potential for generalizing the findings of this study.

## 6 Conclusion and Practice Implications

The evaluation of the PaGODA study indicates that online deliberative forums can achieve high deliberative quality and deliver informed, actionable recommendations from a diverse group of patients as a step towards the development of policy in health data governance. The findings are relevant for managers of genomic data archives, researchers, as well as patient representatives seeking to design, implement, or evaluate participatory governance structures. In particular, the structured and iterative study design—including the follow‐up dialogue event and the integration of the recommendations into a white paper proposing governance procedures of the GHGA—may serve as a transferable model for organizations developing patient involvement policies. While the evaluative steps detailed in this paper are not exhaustive and have limitations, they may support organizations evaluating the success of deliberative processes conducted with the aim of informing, guiding, or implementing policies or other decision‐making processes in various governance domains. Future research should examine the applicability of this model to broader and more diverse patient populations and investigate the longer‐term effects of online deliberation on governance practices and institutional trust.

## Author Contributions

**Apondo Eric:** conceptualization (lead), investigation (lead), writing – original draft (lead), methodology (lead), writing – review and editing (equal), formal analysis (lead), project administration (equal), data curation (lead), supervision (equal), validation (lead), visualization (lead), funding acquisition (supporting). **Schickhardt Christoph:** conceptualization (equal), methodology (supporting), writing – review and editing (equal). **Andrea Züger:** conceptualization (equal), investigation (equal), writing – review and editing (equal), methodology (equal). **Eva C. Winkler:** supervision (lead), resources (lead), conceptualization (equal), writing – review and editing (equal), methodology (equal). **Mehlis Katja:** conceptualization (equal), writing – review and editing (equal), methodology (equal), supervision (lead).

## Ethics Statement

The study protocol was approved by the Ethics Commission of the Medical Faculty of the University of Heidelberg (Ref. S‐061/2022).

## Consent

All participants signed an informed consent prior to participation.

## Conflicts of Interest

The authors declare that the research was conducted in the absence of any commercial or financial relationships that could be construed as a potential conflict of interest.

## Supporting information


Supporting File 1

Supporting File 2

## Data Availability

The raw data supporting the conclusions of this article are included in the Supporting Information. Any further data requested will be made available by the authors, without undue reservation.
