# Supplementary material for: Evaluating the Quality and Impact of Online Patient Forums in Genomic Data Governance
Source: Health Expect. 2026 Jul 30;29(4):e70789. doi: 10.1111/hex.70789 (PMC13421793; doi:10.1111/hex.70789)
Supplement: Supplementary file 1 — Supporting File 1 [file HEX-29-e70789-s002.docx]

| **A. Process design integrity** | | | | | | | | | | | | | | | | | | | | | | | | | | | | | | | | | | | | | | | | | | | | | | | | | | | | | | | | | | | | | | | | | | | | | | | | | | | | | | | | | | | | | | | | | | | | | | | |
| --- | --- | --- | --- | --- | --- | --- | --- | --- | --- | --- | --- | --- | --- | --- | --- | --- | --- | --- | --- | --- | --- | --- | --- | --- | --- | --- | --- | --- | --- | --- | --- | --- | --- | --- | --- | --- | --- | --- | --- | --- | --- | --- | --- | --- | --- | --- | --- | --- | --- | --- | --- | --- | --- | --- | --- | --- | --- | --- | --- | --- | --- | --- | --- | --- | --- | --- | --- | --- | --- | --- | --- | --- | --- | --- | --- | --- | --- | --- | --- | --- | --- | --- | --- | --- | --- | --- | --- | --- | --- | --- | --- | --- | --- | --- | --- |
| **Criteria 1: Suitable design** | | | | | | | | | | | | | | | | | | | | | | | | | | | | | | | | | | | | | | | | | | | | | | | | | | | | | | | | | | | | | | | | | | | | | | | | | | | | | | | | | | | | | | | | | | | | | | | |
| Do you think the length of the process was appropriate? | Yes: 14 (70%)  No, too short: 5 (25%)  No, too long: 1 (5%)  Not sure: 1 (5%) | | | | | | | | | | | | | | | | | | | | | | | | | | | | | | | | | | | | | | | | | | | | | | | | | | | | | | | | | | | | | | | | | | | | | | | | | | | | | | | | | | | | | | | | | | | | | | |
| If you consider the process needed more time, how much more? | 1 day more: 2  At least a day more: 3  At least 2 days: 1 | | | | | | | | | | | | | | | | | | | | | | | | | | | | | | | | | | | | | | | | | | | | | | | | | | | | | | | | | | | | | | | | | | | | | | | | | | | | | | | | | | | | | | | | | | | | | | |
| If you consider the process needed more time, how would you use the extra time? | Presentations from the invited experts: 1  Presentations from other expersts: 2  General discussions: 10  Discussing the recommendations: 7  More breaks: 2 | | | | | | | | | | | | | | | | | | | | | | | | | | | | | | | | | | | | | | | | | | | | | | | | | | | | | | | | | | | | | | | | | | | | | | | | | | | | | | | | | | | | | | | | | | | | | | |
| Do you think the time was well used to arrive at the final recommendations? (0 means”not at all”, 10 means “extremely well used") | 0 | 1 | | | | | | | | 2 | | | | | | | | 3 | | | | | | | | 4 | | | | | | | 5 | | | | | | | | | 6 | | | | | | | | | | | 7 | | | | | | | | 8 | | | | | | | | | 9 | | | | | | | | | 10 | | | | | | | | | Median | | | | | | | |
|  | 0 | 0 | | | | | | | | 0 | | | | | | | | 2(9%) | | | | | | | | 0 | | | | | | | 3(14%) | | | | | | | | | 2(9%) | | | | | | | | | | | 4 (19%) | | | | | | | | 9 (42%) | | | | | | | | | 1 (5%) | | | | | | | | | 0 | | | | | | | | | 7 | | | | | | | |
| How did you experience the balance between time spent in small group discussions and in plenary? | Too much time in small groups, too little in plenary: 2 (11%)  Too much time in plenary too little in small groups: 5 (26%)  The balance was just right: 12 (63%) | | | | | | | | | | | | | | | | | | | | | | | | | | | | | | | | | | | | | | | | | | | | | | | | | | | | | | | | | | | | | | | | | | | | | | | | | | | | | | | | | | | | | | | | | | | | | | |
| **Criteria 2: Representativeness and inclusiveness** | | | | | | | | | | | | | | | | | | | | | | | | | | | | | | | | | | | | | | | | | | | | | | | | | | | | | | | | | | | | | | | | | | | | | | | | | | | | | | | | | | | | | | | | | | | | | | | |
| How many of the other members had different views compared to your own? | None: 2 (9%)  A few: 9 (42%)  About a half: 4 (19%)  Most: 4 (19%)  I don’t know: 2 (9%) | | | | | | | | | | | | | | | | | | | | | | | | | | | | | | | | | | | | | | | | | | | | | | | | | | | | | | | | | | | | | | | | | | | | | | | | | | | | | | | | | | | | | | | | | | | | | | |
| Did you feel there were any groups **not** represented? | Yes: 4 (19%)  No: 12 (57%)  No answer: 5 (23%) | | | | | | | | | | | | | | | | | | | | | | | | | | | | | | | | | | | | | | | | | | | | | | | | | | | | | | | | | | | | | | | | | | | | | | | | | | | | | | | | | | | | | | | | | | | | | | |
| **B. The deliberative experience** | | | | | | | | | | | | | | | | | | | | | | | | | | | | | | | | | | | | | | | | | | | | | | | | | | | | | | | | | | | | | | | | | | | | | | | | | | | | | | | | | | | | | | | | | | | | | | | |
| **Criteria 1: Neutrality and inclusivity of facilitation** | | | | | | | | | | | | | | | | | | | | | | | | | | | | | | | | | | | | | | | | | | | | | | | | | | | | | | | | | | | | | | | | | | | | | | | | | | | | | | | | | | | | | | | | | | | | | | | |
| To what extent do you feel the facilitators were neutral or biased? (O means “completely neutral”; 10 means “very biased”) | 0 | | 1 | | | | | | | | 2 | | | | | | | | 3 | | | | | | | | | 4 | | | | | | | | 5 | | | | | | | | | 6 | | | | | | | | | 7 | | | | | | | | 8 | | | | | | | | | 9 | | | | | | | | | 10 | | | | | | | | | Median | | | | | | |
|  | 8 | | 2 | | | | | | | | 1 | | | | | | | | 2 | | | | | | | | | 0 | | | | | | | | 0 | | | | | | | | | 0 | | | | | | | | | 0 | | | | | | | | 0 | | | | | | | | | 1 | | | | | | | | | 2 | | | | | | | | | 1 | | | | | | |
| **Criteria 2: Breadth, diversity, clarity, and relevance of the evidence and stakeholders** | | | | | | | | | | | | | | | | | | | | | | | | | | | | | | | | | | | | | | | | | | | | | | | | | | | | | | | | | | | | | | | | | | | | | | | | | | | | | | | | | | | | | | | | | | | | | | | |
| Do you feel the information resources provided to you to help (0 means “not sufficient”, 5 “just right” 10 “too mucht information”)  discussions were narrow or broad? | 0 | | 1 | | | | | | | | 2 | | | | | | | | 3 | | | | | | | | | 4 | | | | | | | | 5 | | | | | | | | | 6 | | | | | | | | | 7 | | | | | | | | 8 | | | | | | | | | 9 | | | | | | | | | 10 | | | | | | | | | Median | | | | | | |
|  | 0 | | 0 | | | | | | | | 3 (16%) | | | | | | | | 1 (6%) | | | | | | | | | 1 (6%) | | | | | | | | 10 (56%) | | | | | | | | | 1 (6%) | | | | | | | | | 0 | | | | | | | | 1(6%) | | | | | | | | | 0 | | | | | | | | | 1 (6%) | | | | | | | | | 5 | | | | | | |
| Do you feel that the information resources provided, as a whole were neutral, with fair and diverse viewpoints represented? | 0 | | | 1 | | | | | | | | 2 | | | | | | | | 3 | | | | | | | | | 4 | | | | | | | | 5 | | | | | | | | | 6 | | | | | | | | | 7 | | | | | | | | 8 | | | | | | | | | 9 | | | | | | | | | 10 | | | | | | | | | Median | | | | | |
|  | 0 | | | 0 | | | | | | | | 0 | | | | | | | | 1 (5%) | | | | | | | | | 3 (16%) | | | | | | | | 11 (58%) | | | | | | | | | 1 (5%) | | | | | | | | | 0 | | | | | | | | 1 (5%) | | | | | | | | | 1 (5%) | | | | | | | | | 1 (%) | | | | | | | | | 5 | | | | | |
| Did you find the evidence that was presented by the speakers easy or hard to understand? | I understood the presentation well right from the beginning: 17 (89%)  The presentations were difficult to understand at the beginning, but got easier as the forums progressed: 2 (10%)  The entirety of the presentations was difficult to understand: 0  I don’t know: 0  No answer: 0 | | | | | | | | | | | | | | | | | | | | | | | | | | | | | | | | | | | | | | | | | | | | | | | | | | | | | | | | | | | | | | | | | | | | | | | | | | | | | | | | | | | | | | | | | | | | | | |
| **Criteria 3: Quality of judgement** | | | | | | | | | | | | | | | | | | | | | | | | | | | | | | | | | | | | | | | | | | | | | | | | | | | | | | | | | | | | | | | | | | | | | | | | | | | | | | | | | | | | | | | | | | | | | | | |
| Do you feel that the issue was discussed from a variety of perspectives (for example, considering underlying issues, existing structures, trade-offs values etc.)? (0 means “limited number of perspectives”, 10 means “wide range of perspectives”) | 0 | | | | 1 | | | | | | | | 2 | | | | | | | | 3 | | | | | | | | | 4 | | | | | | | | 5 | | | | | | | | | 6 | | | | | | | | | 7 | | | | | | | | 8 | | | | | | | | | 9 | | | | | | | | | 10 | | | | | | | | | Median | | | | |
|  | 0 | | | | 0 | | | | | | | | 0 | | | | | | | | 0 | | | | | | | | | 2 (11%) | | | | | | | | 5(26%) | | | | | | | | | 2 (11%) | | | | | | | | | 4 (21%) | | | | | | | | 2 (11%) | | | | | | | | | 1 (5%) | | | | | | | | | 3 (15%) | | | | | | | | | 7 | | | | |
| Do you feel that most members were providing justifications and explanations for their opinions? (0 means “most never provided justifications”, 10 means “most provided justifications”) | 0 | | | | | 1 | | | | | | | | 2 | | | | | | | | 3 | | | | | | | | | 4 | | | | | | | | 5 | | | | | | | | | 6 | | | | | | | | 7 | | | | | | | | | 8 | | | | | | | | | 9 | | | | | | | | | 10 | | | | | | | | | Median | | | |
|  | 0 | | | | | 0 | | | | | | | | 0 | | | | | | | | 2 (11%) | | | | | | | | | 1 (5%) | | | | | | | | 2 (11%) | | | | | | | | | 3 (15%) | | | | | | | | 0 | | | | | | | | | 4 (21%) | | | | | | | | | 3 (15%) | | | | | | | | | 4 (21%) | | | | | | | | | 8 | | | |
| **Criteria 4: Perceived knowledge gains by members (Answers on a scale of 0 to 10, 0 means “not at all”, 10 means “to a great extent”).**  **To what extent do you feel that:** | | | | | | | | | | | | | | | | | | | | | | | | | | | | | | | | | | | | | | | | | | | | | | | | | | | | | | | | | | | | | | | | | | | | | | | | | | | | | | | | | | | | | | | | | | | | | | | |
| your understanding of the issue became clearer throughout the process? | 0 | | | | | | 1 | | | | | | | | 2 | | | | | | | | 3 | | | | | | | | | 4 | | | | | | | | 5 | | | | | | | | | 6 | | | | | | | | 7 | | | | | | | | | 8 | | | | | | | | | 9 | | | | | | | | | 10 | | | | | | | | | Median | | |
|  | 0 | | | | | | 1(5%) | | | | | | | | 0 | | | | | | | | 1(5%) | | | | | | | | | 0 | | | | | | | | 1(5%) | | | | | | | | | 0 | | | | | | | | 2(11%) | | | | | | | | | 2(11%) | | | | | | | | | 6 (32%) | | | | | | | | | 6 (32%) | | | | | | | | | 9 | | |
| you gained more arguments and perspectives to support your own opinion about the issue? | 0 | | | | | | 1 | | | | | | | | 2 | | | | | | | | 3 | | | | | | | | | 4 | | | | | | | | 5 | | | | | | | | | 6 | | | | | | | | 7 | | | | | | | | | 8 | | | | | | | | | 9 | | | | | | | | | 10 | | | | | | | | | Median | | |
|  | 0 | | | | | | 1(5%) | | | | | | | | 1(5%) | | | | | | | | 0 | | | | | | | | | 0 | | | | | | | | 4(21%) | | | | | | | | | 3(16%) | | | | | | | | 4(21%) | | | | | | | | | 2(11%) | | | | | | | | | 0 | | | | | | | | | 4(21%) | | | | | | | | | 7 | | |
| you understood the arguments, perspectives, and concerns of others? | 0 | | | | | | 1 | | | | | | | | 2 | | | | | | | | 3 | | | | | | | | | 4 | | | | | | | | 5 | | | | | | | | | 6 | | | | | | | | 7 | | | | | | | | | 8 | | | | | | | | | 9 | | | | | | | | | 10 | | | | | | | | | Median | | |
|  | 0 | | | | | | 0 | | | | | | | | 0 | | | | | | | | 0 | | | | | | | | | 2 (11%) | | | | | | | | 0 | | | | | | | | | 1(5%) | | | | | | | | 1(5%) | | | | | | | | | 5(26%) | | | | | | | | | 3(16%) | | | | | | | | | 7 (36%) | | | | | | | | | 9 | | |
| your understanding of others’ opinions of the issue became clearer through this process? | 0 | | | | | | 1 | | | | | | | | 2 | | | | | | | | 3 | | | | | | | | | 4 | | | | | | | | 5 | | | | | | | | | 6 | | | | | | | | 7 | | | | | | | | | 8 | | | | | | | | | 9 | | | | | | | | | 10 | | | | | | | | | Median | | |
|  | 0 | | | | | | 1(6%) | | | | | | | | 0 | | | | | | | | 1(6%) | | | | | | | | | 1(6%) | | | | | | | | 4(22%) | | | | | | | | | 0 | | | | | | | | 3(17%) | | | | | | | | | 4(22%) | | | | | | | | | 2(11%) | | | | | | | | | 2(11%) | | | | | | | | | 7 | | |
| **Criteria 5: To what extent do you feel that you well informed about the following subjects? (Answers on a scale of 0 to 10, 0 means “not at all”, 10 means “to a great extent”)** | | | | | | | | | | | | | | | | | | | | | | | | | | | | | | | | | | | | | | | | | | | | | | | | | | | | | | | | | | | | | | | | | | | | | | | | | | | | | | | | | | | | | | | | | | | | | | | |
| Informed consent as pertains to genomic (research) data | 0 | | | | | | | 1 | | | | | | | | 2 | | | | | | | | 3 | | | | | | | | | 4 | | | | | | | | 5 | | | | | | | | | 6 | | | | | | | | 7 | | | | | | | | | 8 | | | | | | | | | 9 | | | | | | | | | 10 | | | | | | | | | Median | |
|  | 0 | | | | | | | 0 | | | | | | | | 0 | | | | | | | | 0 | | | | | | | | | 1(6%) | | | | | | | | 1(6%) | | | | | | | | | 1(6%) | | | | | | | | 0 | | | | | | | | | 1(6%) | | | | | | | | | 3(17%) | | | | | | | | | 11(61%) | | | | | | | | | 10 | |
| Data sharing | 0 | | | | | | | 1 | | | | | | | | 2 | | | | | | | | 3 | | | | | | | | | 4 | | | | | | | | 5 | | | | | | | | | 6 | | | | | | | | 7 | | | | | | | | | 8 | | | | | | | | | 9 | | | | | | | | | 10 | | | | | | | | | Median | |
|  | 0 | | | | | | | 0 | | | | | | | | 0 | | | | | | | | 0 | | | | | | | | | 1(6%) | | | | | | | | 1(6%) | | | | | | | | | 0 | | | | | | | | 1(6%) | | | | | | | | | 2(11%) | | | | | | | | | 3(17%) | | | | | | | | | 10(56%) | | | | | | | | | 10 | |
| Governance structures of genomics data archives | 0 | | | | | | | 1 | | | | | | | | 2 | | | | | | | | 3 | | | | | | | | | 4 | | | | | | | | 5 | | | | | | | | | 6 | | | | | | | | 7 | | | | | | | | | 8 | | | | | | | | | 9 | | | | | | | | | 10 | | | | | | | | | Median | |
|  | 0 | | | | | | | 0 | | | | | | | | 0 | | | | | | | | 1(16%) | | | | | | | | | 0 | | | | | | | | 1(16%) | | | | | | | | | 2(11%) | | | | | | | | 0 | | | | | | | | | 6(33%) | | | | | | | | | 3(17%) | | | | | | | | | 5(28%) | | | | | | | | | 8 | |
| **Criteria 6: Accessibility and equality of opportunity to speak. To what extent do you feel (0 means “absolutely disagree”, 10 means “absolutely agree”):** | | | | | | | | | | | | | | | | | | | | | | | | | | | | | | | | | | | | | | | | | | | | | | | | | | | | | | | | | | | | | | | | | | | | | | | | | | | | | | | | | | | | | | | | | | | | | | | |
| you had a fair number of opportunities to speak? | 0 | | | | | | | | 1 | | | | | | | | 2 | | | | | | | | 3 | | | | | | | | | 4 | | | | | | | | 5 | | | | | | | | | 6 | | | | | | | | 7 | | | | | | | | | 8 | | | | | | | | | 9 | | | | | | | | | 10 | | | | | | | | | Median |
|  | 0 | | | | | | | | 0 | | | | | | | | 0 | | | | | | | | 0 | | | | | | | | | 0 | | | | | | | | 0 | | | | | | | | | 0 | | | | | | | | 1(16%) | | | | | | | | | 4(22%) | | | | | | | | | 2(11%) | | | | | | | | | 11(61%) | | | | | | | | | 10 |
| other members had a fair number of opportunities to speak? | 0 | | | | | | | | 1 | | | | | | | | 2 | | | | | | | | 3 | | | | | | | | | 4 | | | | | | | | 5 | | | | | | | | | 6 | | | | | | | | 7 | | | | | | | | | 8 | | | | | | | | | 9 | | | | | | | | | 10 | | | | | | | | | Median |
|  | 0 | | | | | | | | 0 | | | | | | | | 0 | | | | | | | | 0 | | | | | | | | | 0 | | | | | | | | 1(16%) | | | | | | | | | 1(16%) | | | | | | | | 1(16%) | | | | | | | | | 3(17%) | | | | | | | | | 2(11%) | | | | | | | | | 10 (56%) | | | | | | | | | 10 |
| all members were heard equally? | 0 | | | | | | | | 1 | | | | | | | | 2 | | | | | | | | 3 | | | | | | | | | 4 | | | | | | | | 5 | | | | | | | | | 6 | | | | | | | | 7 | | | | | | | | | 8 | | | | | | | | | 9 | | | | | | | | | 10 | | | | | | | | | Median |
|  | 0 | | | | | | | | 0 | | | | | | | | 0 | | | | | | | | 0 | | | | | | | | | 1(16%) | | | | | | | | 0 | | | | | | | | | 1(16%) | | | | | | | | 1(16%) | | | | | | | | | 4(22%) | | | | | | | | | 1(16%) | | | | | | | | | 9(50%) | | | | | | | | | 10 |
| some members dominated the small group discussions? | 0 | | | | | | | | 1 | | | | | | | | 2 | | | | | | | | 3 | | | | | | | | | 4 | | | | | | | | 5 | | | | | | | | | 6 | | | | | | | | 7 | | | | | | | | | 8 | | | | | | | | | 9 | | | | | | | | | 10 | | | | | | | | | Median |
|  | 2(11%) | | | | | | | | 1(6%) | | | | | | | | 1(6%) | | | | | | | | 2(11%) | | | | | | | | | 0 | | | | | | | | 1(6%) | | | | | | | | | 1(6%) | | | | | | | | 3(18%) | | | | | | | | | 2(11%) | | | | | | | | | 3(18%) | | | | | | | | | 1(6%) | | | | | | | | | 7 |
| you and your views were heard? | 0 | | | | | | | | 1 | | | | | | | | 2 | | | | | | | | 3 | | | | | | | | | 4 | | | | | | | | 5 | | | | | | | | | 6 | | | | | | | | 7 | | | | | | | | | 8 | | | | | | | | | 9 | | | | | | | | | 10 | | | | | | | | | Median |
|  | - | | | | | | | | - | | | | | | | | - | | | | | | | | - | | | | | | | | | - | | | | | | | | - | | | | | | | | | 1 (5%) | | | | | | | | - | | | | | | | | | 2 (11%) | | | | | | | | | 5 (26%) | | | | | | | | | 9 (47%) | | | | | | | | | 10 |
| **Criteria 7: Respect and mutual comprehension** | | | | | | | | | | | | | | | | | | | | | | | | | | | | | | | | | | | | | | | | | | | | | | | | | | | | | | | | | | | | | | | | | | | | | | | | | | | | | | | | | | | | | | | | | | | | | | | |
| To what extent do you feel that fellow members respected what you had to say, even when they didn't agree with you? | 0 | | | | | | | | 1 | | | | | | | | | 2 | | | | | | | | | 3 | | | | | | | | 4 | | | | | | | | | 5 | | | | | | | | 6 | | | | | | | | 7 | | | | | | | | | 8 | | | | | | | | | 9 | | | | | | | | | 10 | | | | | | | | Median |
|  | 0 | | | | | | | | 0 | | | | | | | | | 0 | | | | | | | | | 0 | | | | | | | | 0 | | | | | | | | | 0 | | | | | | | | 0 | | | | | | | | 1(6%) | | | | | | | | | 5(31%) | | | | | | | | | 4(25%) | | | | | | | | | 6(38%) | | | | | | | | 9 |
| **C: Pathways to impact** | | | | | | | | | | | | | | | | | | | | | | | | | | | | | | | | | | | | | | | | | | | | | | | | | | | | | | | | | | | | | | | | | | | | | | | | | | | | | | | | | | | | | | | | | | | | | | | |
| Imagine you are the decision-maker that convened this process. Would you implement the recommendations the deliberative process produced? | | | | | | | | | | | | | | | | | | | | | | | | | | | | | | | | | | | | | | | | | | | Yes, all of them: 0  Yes, most (>75%): 14  Yes, about 50%: 2  Yes, some (25-50%): 2  No: 0 | | | | | | | | | | | | | | | | | | | | | | | | | | | | | | | | | | | | | | | | | | | | | | | | | | | | |
| Does the outcome of the deliberative process match your expectations? | | | | | | | | | | | | | | | | | | | | | | | | | | | | | | | | | | | | | | | | | | | Yes: 21/24 (87,5%)  No answer: 3 | | | | | | | | | | | | | | | | | | | | | | | | | | | | | | | | | | | | | | | | | | | | | | | | | | | | |
| What form should patient involvement in genomic data archives take? | | | | | | | | | | | | | | | | | | | | | | | | | | | | | | | | | | | | | | | | | | | -Involvement of patient representatives in existing boards: 14  -Establishing separate patient advisory boards: 8  Other: 1 | | | | | | | | | | | | | | | | | | | | | | | | | | | | | | | | | | | | | | | | | | | | | | | | | | | | |
